# Mating status affects female choice when females are signalers

**DOI:** 10.1002/ece3.8864

**Published:** 2022-04-19

**Authors:** Naomi L. Zweerus, Michiel van Wijk, Isabel M. Smallegange, Astrid T. Groot

**Affiliations:** ^1^ 1234 Institute for Biodiversity and Ecosystem Dynamics University of Amsterdam Amsterdam Netherlands; ^2^ 5994 School of Natural & Environmental Sciences Newcastle University Newcastle upon Tyne UK

**Keywords:** *Chloridea*(*Heliothis*) *virescens*, female mate choice, female sexual behavior, mating status, multiple mating, signaling

## Abstract

Sexual selection in animals has been mostly studied in species in which males are signalers and females are choosers. However, in many species, females are (also) signalers. In species with non‐signaling females, virgin females are hypothesized to be less choosy than mated females, as virgins must mate to realize fitness and the number of available males is generally limited. Yet, when females signal to attract males, mate limitation can be overcome. We tested how virgin and mated females differ in their calling behavior, mating latency, and in mate choice, using the tobacco budworm *Chloridea* (*Heliothis*) *virescens* as an example for a species in which females are not only choosers but also signalers. We found that virgin females signaled longer than mated females, but virgin and mated signaling females were equally ready to mate, in contrast to non‐signaling females. However, we found that virgin signaling females showed weaker mate preference than mated females, which can be explained by the fact that females increase their fitness with multiple matings. Mated females may thus further increase their fitness by more stringent mate selection. We conclude that signaling is a crucial aspect to consider when studying female mate choice because signaling may affect the number of available mates to choose from.

## Graphical abstract

When females signal to attract males, mate limitation can be overcome allowing all females to be choosy. In this study, we tested if female signaling and mate choice differs between virgin and mated females. While virgin females signaled more, virgin and mated signaling females were equally ready to mate but virgin signaling females showed weaker mate preference than mated females.

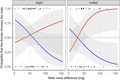

## 1 INTRODUCTION

Female mating decisions are influenced by her mating history (Jennions & Petrie, 1997; Kelly, 2018). When females mate multiple times during their lifetime, their mate choice may depend on whether they are still virgin or have already mated (Jennions & Petrie, 1997) because each mating status has different costs and benefits (Halliday, 1983; Herberstein et al., 2002; Kelly, 2018; Kokko & Mappes, 2005). Virgins need to mate to realize fitness (Wickman & Rutowski, 1999) and should thus mate with any male (Jennions & Petrie, 2000; Kokko & Mappes, 2005). Once mated, females can benefit from additional matings (Jennions & Petrie, 2000; Parker & Birkhead, 2013; Puurtinen & Fromhage, 2017; Zeh & Zeh, 2001) because these can provide nutritional or immune boosting nuptial gifts (Brown, 1997; Vahed, 1998; Worthington & Kelly, 2016), compensate for previous, infertile matings (Sheldon, 1994), and provide access to a larger gene pool (genetic benefits, Thornhill, 1983). Multiple matings may also allow females to store sperm from multiple partners and select for paternity (cryptic female choice, Eberhard, 1996; Thornhill, 1984). The genetic make‐up of offspring depends on whether the first or the last mated male sires all offspring (first and last male sperm precedence), or whether paternity varies (variable sperm precedence) (Birkhead & Hunter, 1990; Parker, 1970; Simmons, 2001). The number of matings and the pattern of sperm precedence can thus affect how much effort a female allocates to precopulatory mate assessment and directs mate choice.

Virgin females and mated females may differ in their level of choosiness because her sexual behavior may change once sperm for fertilization is obtained (Jennions & Petrie, 2000; Kokko & Mappes, 2005). For non‐signaling females, theory predicts that virgins are less choosy than mated females (Jennions & Petrie, 2000; Kokko & Mappes, 2005). Choosiness can be seen as the effort made by the female to choose a male (Edward, 2015; Jennions & Petrie, 1997; Widemo & Sæther, 1999), which can be reflected in the time until mating (mating latency) (Holveck & Riebel, 2010; Lindström & Lehtonen, 2013), and the strength of mate preference (i.e., the slope of the preference function (Brooks & Endler, 2001; Cotton et al., 2006; Ratterman et al., 2014) or height of the preference function (Kilmer et al., 2017)). For instance, in guppies (*Poecilia reticulata*) and field crickets (*Gryllus bimaculatus*), virgin females show weaker mate preference than mated females (Bateman et al., 2001; Pitcher et al., 2003). Also, virgin female red flour beetles (*Tribolium castaneum*) choose lower‐quality males than mated females (Fedina & Lewis, 2008). Such a difference in choosiness between virgin and mated females is expected to be amplified in systems with last–male sperm precedence because the last male sires all offspring and thus, only the last choice matters (Kokko & Mappes, 2005). A large difference in choosiness between virgin and mated females is indeed seen in female smooth newts (*Titurus vulgaris*) (Gabor & Halliday, 1997) and fall field crickets *Gryllus pennsylvanicus* (Judge et al., 2010), which are examples of species with last‐male sperm precedence.

The chance of mating may not only increase with a low level of choosiness in virgin females but also with active signaling by females. Through signaling, females attract males and thus affect male availability. In the extreme case of non‐flying females, like glow‐worms (Baudry et al., 2021; Elgert et al., 2021; Hopkins et al., 2015; South et al., 2011), praying mantids (Maxwell et al., 2010), or wingless moths (e.g., Roelofs et al., 1982; Wong et al., 1984), signaling is often crucial to secure a mate. The more males a female attracts, the higher her chance for mating and the more options to choose a mate. When signaling attracts males of different quality, females may directly compare males and choose for relatively higher‐quality partners (reviewed in Jennions & Petrie, 1997). The level of signaling and choosiness are thus crucial elements of female reproductive strategies.

In non‐signaling females, female behavior has no effect on how many males she can choose from, which likely has consequences for her choosiness. Non‐signaling females may mate with any arriving male because there may not be other (additional males) coming in her vicinity, and mating is probably better than remaining unmated. Interestingly, while theory on non‐signaling females predicts that virgins should be less choosy than mated females (Jennions & Petrie, 2000; Kokko & Mappes, 2005), theoretical predictions for virgin and mated female reproductive strategies for signaling females are, to our knowledge, lacking. Such predictions are important to understand the evolution of female signaling and choosiness.

In this study, we determined how signaling differs between virgin and mated females and assessed whether predictions from non‐signaling females (i.e., that virgins are less choosy than mated females) hold for signaling females. Female tobacco budworm *Chloridea* (*Heliothis*) *virescens* (Lepidoptera: Noctuidae) are an ideal study system because these female moths signal and also choose their mating partners (Zweerus et al., 2021). Also, decades of research on this species provides much information on its mating behavior. Females signal by everting their pheromone gland from the tip of the abdomen, a behavior termed “calling”, which releases the species‐specific sex pheromone to attract males (Löfstedt, 1993; Tumlinson et al., 1975). Female signaling and male responses are synchronized within a specific window of time at night (reviewed in Groot, 2014; Phelan, 1997). Under laboratory conditions, *C*. *virescens* females start signaling about half‐way into the scotophase (i.e., the dark period of the diel cycle), peaking at around 6 h into the dark under 14:10 L:D conditions (Heath et al., 1991; Pope et al., 1982; NLZ pers. observation). Both males and females mate only once per night, but several times over multiple nights (Flint & Kressin, 1968; Gao et al., 2020; Pair et al., 1977; Raulston et al., 1975). Previous research showed that females choose mates during courtship and virgin *C*. *virescens* females prefer to mate with larger, higher‐quality males (Zweerus et al., 2021). Matings generally last 2–3 h (Blanco et al., 2009; Hosseini et al., 2016; Pair et al., 1977), during which the male transfers a spermatophore (Blanco et al., 2009; LaMunyon, 2000). Even though one spermatophore can provide enough sperm to inseminate all eggs (LaMunyon, 2000), females mate multiple times (Blanco et al., 2009; Raulston et al., 1975). In multiple‐mated *C*. *virescens* females, sperm precedence is variable (Blanco et al., 2008; LaMunyon, 2000; LaMunyon & Eisner, 1993). Mated females gain fitness with additional matings as their offspring number increases with every mating, peaking after three matings (Gao et al., 2020).

To test the effect of mating status on sexual behavior and mate choice, we conducted three experiments on virgin and mated *C*. *virescens* females. First, we evaluated whether virgin females and mated females differ in their signaling effort. Since the necessity to mate is higher in virgin females than in mated females, we expected virgin females to signal more or for longer than mated females. Second, we tested the hypothesis that virgin females have a shorter mating latency (as a proxy of readiness to mate) than mated females, again because virgins must secure a mate. Finally, we tested if virgin females show a weaker preference for larger males than mated females because less stringent selection of males creates a higher chance for mating.

## 2 METHODS

### 2.1 Study organism

*Chloridea*(*Heliothis*) *virescens* populations originated from North Carolina State University (YDK strain) and the Max Planck Institute for Chemical Ecology, Jena, and have been reared at the Institute for Biodiversity and Ecosystem and Dynamics (IBED), University of Amsterdam, since 2011. We conducted the experiment for this study between April 2018 and November 2019. The moths were kept in a climate chamber at 60% relative humidity and 25 ± 1°C, with a 14 h light (photophase): 10 h dark (scotophase) photoperiod (lights off at 11:00 a.m. CET). Larvae were reared on artificial pinto bean diet (Burton, 1970) in individual plastic cups (37 ml, Solo, Lake Forest, Illinois). Pupae were checked daily for eclosion (i.e., hatching of adult) and emerged adults were fed 10% sucrose solution provided through 1 cm cotton dental wick. All experiments were conducted with 2‐ to 3‐day‐old non‐sibling individuals and were conducted under the same environmental conditions as those used for rearing.

### 2.2 Procedure to obtain mated females and maintaining virgin females

To obtain mated females, we placed an adult female (first day after eclosion) in a large clear plastic cup (473 ml, Solo) with an adult male that was randomly chosen from the adults of the standard rearing. At the same time, for the “virgin group”, females were also selected on the first day after eclosion and individually isolated. We next observed all pairs at 30‐min intervals during the scotophase (dark period) until all pairs were mating, or until 9 h of the scotophase had passed. Because *C*. *virescens* matings last for several hours, only females that mated for ≥60 min qualified for the “mated group” of the experiment. In the photophase that followed the scotophase (i.e., light period on day 2), we individually isolated the mated females. We conducted the experiments on the third day after eclosion, under the same environmental conditions as the rearing and the preparatory matings.

### 2.3 Procedure to obtain an extended range of male body sizes

Previously we found that male body size affects female fitness: females mating with larger males had more offspring than when mating with smaller males (Zweerus et al., 2021). To assess female preference for males of different body sizes (a proxy for quality), we increased the range of male sizes by altering the larval diet, following Zweerus et al. (2021). Briefly, we obtained males of average‐to‐large size by rearing larvae on a standard pinto bean diet (Burton, 1970), and obtained males of smaller than average size by rearing larvae on diet whose nutritional value was 25% of the standard diet. It is important to note that even on standard diet, male mass varies. The low nutritional diet extended this “natural” range of masses at the lower end (see Zweerus et al., 2021, Figure S1). The diet treatment was thus a tool to increase the range of male masses without being a factor in the experimental design. For the experiments, we did not use males from one or the other diet, but rather males that differed in pupal mass.

### 2.4 Mate attraction effort experiment

To compare mate attraction between virgin and mated females using their signaling behavior as a proxy of mate attraction effort, we quantified the signaling activity of all females (virgin: *n* = 24 and mated: *n* = 21) in the third scotophase after eclosion as follows. In the hour prior to the scotophase, we placed single females into large clear plastic cups (473 ml, Solo). From the onset of scotophase, we observed and scored the number of females signaling every 15 min. We stopped observing an individual if the female did not signal for at least 1 h. To test if the proportion of signaling females differed between the virgin and mated group, we used a Chi‐square test for independence. To assess if virgin females (*n* = 21) started signaling later than mated females (*n* = 17), we analyzed the normally distributed data with equal variances for the onset time of signaling with a two‐tailed *t*‐test. To test if the duration of signaling differed between virgin and mated females, we used a Mann–Whitney *U*‐test because the data were non‐normally distributed (assessed by Shapiro–Wilk test and visual exploration of histograms). We visualized the data by fitting the proportion of signaling females per time point and mating status over the scotophase using the package ggplot2 (Wickham, 2016) in the software R (version 4.0.5, R Core Team (2021)).

### 2.5 No‐choice experiment to assess readiness to mate in virgin and mated females

To test the hypothesis that virgin females have a shorter mating latency (as a proxy of readiness to mate) than mated females, we measured their mating latency (the time from the pairing until copulation) in no‐choice mating assays. Firstly, we placed one female with one male into a clear plastic cup (473 ml, Solo) and covered the cup with a mesh. We then mounted each plastic cup in a hanging grid with a camera (GoPro Hero silver) underneath. The assay started at the beginning of the scotophase, after which we recorded a time‐lapse series of 1pic/min. We collected the data on minimally 4 and maximally 20 samples per time over eight scotophases between 11th and 20th of May 2018. To determine mating latency, we identified the timestamp of the picture showing a newly formed mating pair in the time‐lapse series. For individuals that did not mate, we censored their data by assigning a maximum time span of 600 min, which corresponds to an entire scotophase. To test if virgin females (*n* = 61) mated significantly earlier than mated females (*n* = 29), and whether male pupal mass or female pupal mass affected mating latency, we fitted a Cox proportional hazards model with the explanatory variables *mating status*, *male pupal mass*, and *female pupal mass* as main effects and mating latency as the response variable in R using the packages survival (Therneau, 2021) and survminer (Kassambara et al., 2019).

### 2.6 Two‐choice experiment to compare virgin and mated female mate preferences

To test the hypothesis that virgin females show a weaker preference for larger (i.e., higher quality) males than mated females, we conducted two‐choice tests, in which we placed one female (virgin: *n* = 61 or mated: *n* = 63) together with a larger and a smaller male into a BugDorm cage (H30 cm × W30 cm × D30 cm), and scored which male a female mated. To distinguish between the two males, we marked each male by clipping the tip of one randomly chosen wing. The experiment started 10 min before the onset of the scotophase and we checked all cages at 30 min intervals. Once a mating pair had formed, we removed the unmated male from the cage. To check if the matings were successful, we isolated and froze all individuals in the next photophase, dissected the females, and quantified the number of spermatophores per individual. We collected the data over a total of 13 scotophases between August 13 and November 8, 2019. Since not all females mated in the two‐choice assay (virgin: *n* = 56, mated: *n* = 45), we first tested for an association between mating status (virgin, mated) and mating occurrence (mating, no mating) with a Fisher's exact test. Additionally, we confirmed that the mass range of larger and smaller males did not differ significantly between males offered to virgin females and to mated females, using a Welch's two‐sample *t*‐test. Finally, we determined if the size range between virgin females and mated females differed using a Welch's two‐sample *t*‐test.

To assess whether the males that females chose to mate with were on average larger than the rejected males, we first tested if mean pupal mass differed significantly between the chosen and not chosen males by computing paired *t*‐tests. To then test whether virgin and mated females differed in the strength of their mate preference, we first randomly selected the data of one male per cage (i.e., this male was either chosen or not chosen by the female). Since each female made one choice, this step ensured that the number of data points for the analysis corresponded to the actual number of choices made in the experiment. Subsequently, we modeled the response variable *female choice* as a function of the variable *larger*/*smaller* (male), indicating whether the male was larger or smaller compared to the other male in the same cage, the *difference in male pupal mass* between the two males per cag*e*, female *mating status*, and their three‐way interaction. Including a three‐way interaction in the model allowed us to let the slopes of the function vary independently, and thus enabled us to identify the differences in female mate choice with respect to female mating status. We fitted the model (glm) with a binomial error distribution and produced the ANOVA table using the package car (Fox & Weisberg, 2019) in R. The results were visualized using the package ggplot2 (Wickham, 2016).

## 3 RESULTS

### 3.1 Mate attraction effort experiment: virgin females signal more, but not earlier, than mated females

**FIGURE 1:**
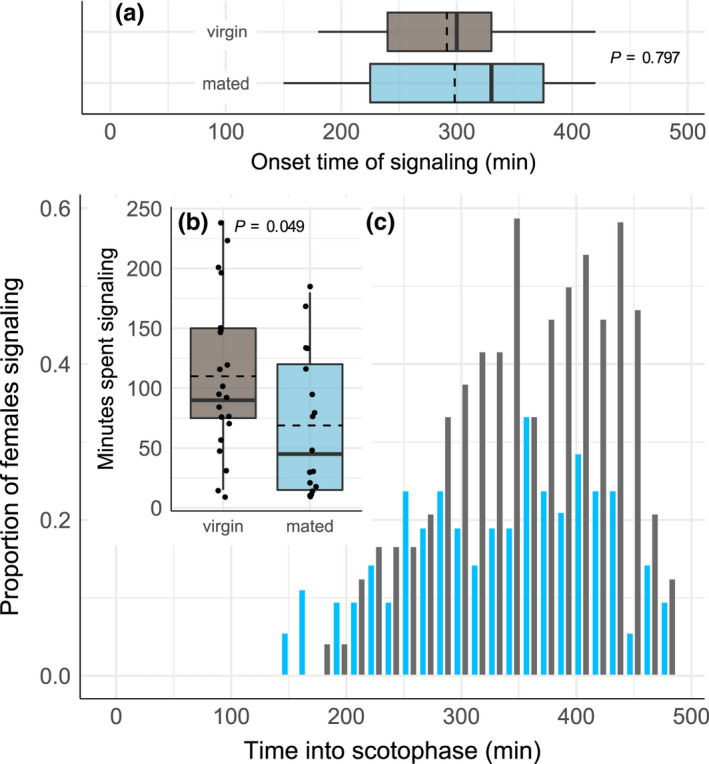
Signaling behavior of virgin females (in grey, *n* = 21) and mated females (in blue, *n* = 17) as (a) onset time of signaling, (b) signaling duration, (c) signaling pattern over time. The upper and lower borders of each box plot indicate the first and third quartile. Thick bars within a box indicate the group median. Dashed lines within a box indicate the group mean. Whiskers above and below each box extend to a maximum of 1.5 times the interquartile range. Dots represent individual data points

There was no significant difference in the proportion of virgin and mated females that signaled in the third scotophase after eclosion (virgin: *n* = 21, 87.5% (21/24), mated: *n* = 17, 80.9% (17/21); *χ* ^2^_1_ = 0, *p* = 1.00). Also, both virgin and mated females started signaling around the same time into the scotophase (virgins: 291.4 ± 14.4 min SE into scotophase, mated: 298.2 ± 21.9 min, *t*‐test: *t* = 0.269, df = 36, *p* = .797) (Figure 1a). However, virgin females spent more time signaling (90 min, IQR 75–150 min) than mated females (45 min, IQR 15–120 min) (*U* = 476.00, *p* = .049) (Figure 1b). Overall, more virgin than mated females signaled per time point (Figure 1c).

### 3.2 No‐choice experiment: virgin and mated females are equally ready to mate

**FIGURE 2:**
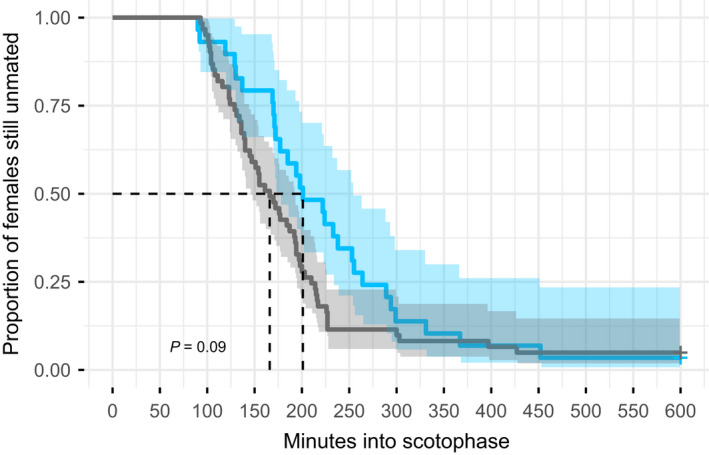
Mating latencies of virgin (in grey) and mated females (in blue), expressed as survival curves over time. Shaded area around each curve: 95% confidence interval. Dashed lines: time point when 50% of the females mated

In the mating latency assay, 86 of 90 pairs (61 virgins and 29 mated females) mated, while 3 virgin females and 1 mated female did not mate. Neither male mass (*χ* ^2^_1_ = 0.49, *p* = .48) nor female mass (*χ* ^2^_1_ = 2.3, *p* = .13) significantly affected mating latency. In contrast to our hypothesis, virgin females did not mate significantly quicker than mated females (log‐rank test: *χ* ^2^_1_ = 2.9, *p* = .09) (Figure 2). Interestingly, however, there was a non‐significant trend toward the predicted direction.

### 3.3 Two‐choice experiment: virgin females are less choosy than mated females

**FIGURE 3:**
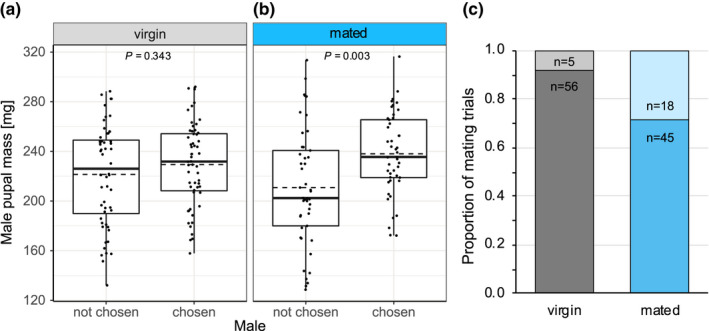
Mass distribution of chosen and non‐chosen males and mating proportions of virgin and mated females. Pupal mass of not chosen and chosen males by (a) virgin (*n* = 56) and (b) mated (*n* = 45) females. Boxplot conventions are as in Figure 1. (c) Proportion of mating events in trials with virgins (grey) and mated females (blue). Dark color = mating, light color = no mating, and *n* = sample size

The ranges of male mass did not significantly differ between the trials involving virgins (132.1–292.0 mg, with a mean of 225.4 ± 3.5 mg SE) and mated (128.6–316.4 mg, with a mean of 224.4 ± 4.5 mg) females (Welch's *t*‐test: *t* = −1.175, df = 177.24, *p* = .861) (Figure 3a,b). In the two‐choice experiments, 91.8% (56/61) of virgin females mated, while only 71.4% (45/63) of mated females mated (Fisher's exact test, *p* = .014) (Figure 3c). Males chosen by virgin females weighed on average 229.4 ± 4.5 mg and the non‐chosen males averaged 221.4 ± 5.4 mg (paired *t*‐test: *t* = −0.958, df = 55, *p* = .343) (Figure 3a). In comparison, males chosen by mated females weighed on average 238.0 ± 4.9 mg, while the non‐chosen males had an average pupal mass of 210.8 ± 7.0 mg (paired *t*‐test: *t* = −3.152, df = 44, *p* = .003) (Figure 3b). Post‐hoc analysis on female size showed that the size range of virgin females (*n* = 56) was 115.2–311.6 mg (mean 223.9 ± 5.4 mg SE) and similar to the size range of mated females (*n* = 45), which was 148.0–293.5 mg (mean 226.7 ± 5.3 mg SE) (*t*‐test: *t* = 0.367, df = 99, *p* = .714).

**FIGURE 4:**
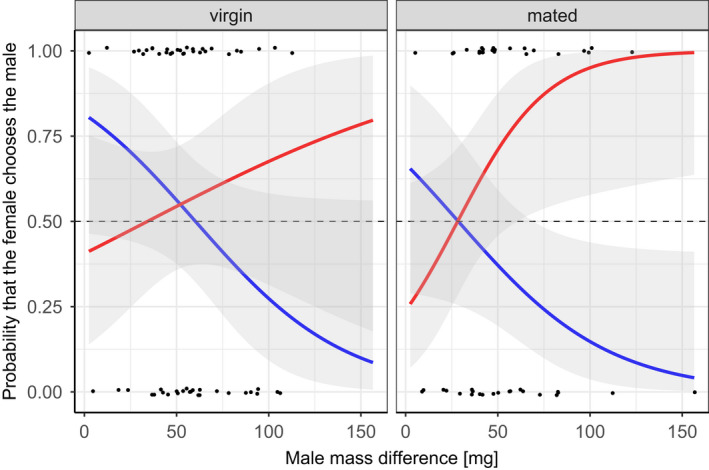
Probability that a virgin (left panel) or mated female (right panel) chooses the relatively larger (red) or smaller male (blue) offered to the female. The curves show model predicted values for male mating probability based on the difference in male mass. Jittered dots: actual data points. Dashed line: 50% mating probability. Shaded area around curves is the 95% CI

Mated females selected more strongly for relatively larger males (Figure 4). In testing the strength of female preference in virgin and mated females, we found a highly significant, three‐way interaction in our model (*χ* ^2^_1_ = 11.244, *p* = .004, Table 1), showing that virgin females (*n* = 56) had a weaker preference than mated females (*n* = 45) (Figure 4). This result thus confirms the hypothesis that virgin females are less choosy than mated females. Moreover, our model revealed that the *relative* male mass significantly affected female choice in mated females (*χ* ^2^_1_ = 4.486, *p* = .034, Table 1). Once the mass difference between the two offered males exceeded 55 mg, mated females differentiated between larger and smaller males, while virgin females did not (Figure 4).

**TABLE 1:** Analysis of deviance table

	*χ* ^2^	Degrees of freedom	*p*‐value
1 Larger/smaller	3.366	1	.067
2 Mass difference	4.486	1	.034
3 Female mating status	1.962	1	.161
Interaction 1:2:3	11.244	2	.004

Note

Model structure: Choice ~ (larger/smaller × mass difference) × mating status + larger/smaller + mass difference + mating status.

## 4 DISCUSSION

In this study, we compared female sexual behavior and mate choice between virgin and mated females in a system where females are not only choosers but also signalers. As expected, we found virgin females to signal longer than mated females. We also found that virgin and mated females showed similar mating latencies, our proxy for readiness to mate. When females could choose between two males, virgin females showed a weaker preference for larger males compared to mated females. Below we discuss these findings in the context of multiple matings.

### 4.1 Signaling behavior

Since virgin females must reduce the risk of remaining unmated, we hypothesized virgin females to signal more or for longer than mated females, which we confirmed in this study. A higher signaling rate of virgin compared to mated females has previously been found in other Heliothine moths, such as the corn earworm *Helicoverpa zea* (Kingan et al., 1993), the subflexus straw *Chloridea (Heliothis) subflexa* (Blankers et al., 2021), and the Pyralid Indian meal moth *Plodia interpunctella* (Brady & Smithwick, 1968). These intuitive findings support the notion that virgin signaling females put more effort into mate attraction effort compared to mated females to increase their chance for mating.

Female mating status had no effect on the onset time of signaling, and we found virgins and mated females to signal within the same time window. Finding overlapping female signaling windows is consistent with species‐specific synchronized signaling, which minimizes communication interference with individuals from closely related species ( Monti et al., 1995; Pashley et al., 1992; Schöfl et al., 2009; Teal et al., 1978; reviewed in Groot, 2014). Even though mated females would have obtained enough sperm from the first mating to fertilize all their eggs (LaMunyon, 2000), we found that about 30% of mated females continued to signal in the night that followed a mating. In the closely related moth *C*. *subflexa*, a lower percentage of females was found calling in the night after mating, however, calling effort increased to about 50% again in subsequent nights (Blankers et al., 2021). The fact that mated females continue to signal emphasizes that both sexes may benefit from mating multiply (Blankers et al., 2021; Gao et al., 2020).

### 4.2 Female readiness to mate (i.e., mating latency)

We found no difference in mating latency between virgin and mated signaling females and thus their readiness to mate, which contrasts the hypothesis that virgin females mate quicker than mated females (Kokko & Mappes, 2005). While a shorter mating latency would be expected in non‐signaling and non‐choosy virgin females, our results suggest that signaling virgin females might be choosy. As females were only offered one male (no‐choice assay), the mating latencies that we measured are possibly the minimal times that females need for mate assessment. Female mate assessment likely starts after 120 min into scotophase because both virgin and mated females only start to become sexually active after this time point, as reflected by their signaling behavior (see Figure 1). It must be noted here that context does affect the timing of sexual activity, as female sexual activity started earlier when a male was present than when females were alone in the signaling assay, even though the cups used in these experiments were identical. Matings occurred throughout the entire period in which females signaled. The fact that we found virgin and mated signaling females equally ready to mate further makes sense because multiple matings increase female fitness in this species (Gao et al., 2020).

### 4.3 Female mate preference

In line with the hypothesis that virgin females have a weaker mate preference than mated females, we found that virgin females had a weaker preference for larger males than mated females. This finding is consistent with predictions for non‐signaling females where virgins are expected to be less choosy (Kokko & Mappes, 2005). However, these predictions did not take into account that signaling may result in attracting multiple males. Apparently, signaling is a factor that allows virgin females to be choosy, although not to the same extent as mated females, which may be due to the fact that virgin females are constrained by their necessity to mate. Once a first mating has been secured, *C*. *virescens* females can increase their fitness by stronger selection for high‐quality males.

Whereas the most obvious effect of the reduced diet was a reduction in male pupal mass, other effects of the reduced diet on male phenotypes cannot be excluded. In our experiments, we manipulated the difference in body mass because we already discovered that this variable affects female choice (Zweerus et al., 2021). However, we cannot exclude that diet affected additional traits unknown to us that contributed to female choice.

### 4.4 Future perspectives: mate choice of polygamous females

Recently, Gao et al. (2020) discovered that *C*. *virescens* females reach maximal fitness with three matings, which suggests that females should maintain a (low) level of choosiness to increase their mating chances until they have reached their mating “optimum” (Gao et al., 2020). However, because female fitness declines after three matings (Gao et al., 2020), thrice‐mated females may become choosier, potentially to such an extent that these females will not mate if no male meets their mate acceptance threshold (De Jong & Sabelis, 1991). To unravel how females optimize their fitness, it would thus be interesting to assess whether mate preferences become stronger when females mate more than twice, and how sequential mate choice translates into paternity (see also Kokko et al., 2006; Slatyer et al., 2012).

Since *C*. *virescens* females mate multiply to increase fitness, mated females may “trade‐up” in partner quality when they remate (Halliday, 1983; Jennions & Petrie, 2000). Such a trading up further increases (genetic) benefits and maximizes fitness. For example, in guppies, females not only remate with higher‐quality males but their eggs are also more likely to be fertilized with sperm from the higher‐quality male (Pitcher et al., 2003). In *C*. *virescens*, females can perhaps bias paternity toward higher‐quality males to increase the genetic benefits for their offspring. The fact that sperm precedence in *C*. *virescens* is variable (LaMunyon, 2000, 2001) suggests that cryptic female choice may occur in this species.

### 4.5 Female mate choice in signaling females compared to non‐signaling females

In conclusion, our results show that in a species where females are signalers as well as choosers, virgin females are choosy too. This differs from the general idea that virgin females mate unselectively, which may be the case in species without signaling females. Non‐signaling females cannot affect mate availability, and virgin females are probably more likely to mate with any available male and thus less choosy than mated females to decrease the risk of remaining unmated. In contrast, signaling allows virgin females to be choosy because signaling females can affect the arrival and number of available males. Since multiple males may be attracted, both virgin and mated females likely benefit from choosing the best male. This explains our finding that virgin and mated females were equally ready to mate. We propose that female signaling should be considered as a crucial component of female mate choice and taken into account to understand the evolution of female choice.

## CONFLICT OF INTEREST

None declared.

## AUTHOR CONTRIBUTIONS

**Naomi L. Zweerus:**Conceptualization (equal); Data curation (lead); Formal analysis (lead); Investigation (equal); Methodology (equal); Project administration (equal); Software (equal); Validation (equal); Visualization (equal); Writing – original draft (lead); Writing – review & editing (equal). **Michiel van Wijk:** Conceptualization (supporting); Data curation (supporting); Formal analysis (equal); Investigation (supporting); Methodology (equal); Software (equal); Validation (equal); Visualization (equal); Writing – original draft (supporting); Writing – review & editing (supporting). **Isabel M. Smallegange:** Conceptualization (equal); Formal analysis (supporting); Investigation (supporting); Methodology (supporting); Visualization (supporting); Writing – original draft (equal); Writing – review & editing (equal). **Astrid T. Groot:** Conceptualization (lead); Formal analysis (supporting); Funding acquisition (lead); Investigation (equal); Methodology (equal); Project administration (lead); Resources (lead); Supervision (lead); Visualization (equal); Writing – original draft (equal); Writing – review & editing (lead).

## Data Availability

Data sets are publicly available at Data Dryad https://doi.org/10.5061/dryad.8cz8w9gsx.

## References

[ece38864-bib-0001] Bateman, P. W. , Gilson, L. N. , & Ferguson, J. (2001). Male size and sequential mate preference in the cricket *Gryllus bimaculatus* . Animal Behaviour, 61(3), 631–637. 10.1006/anbe.2000.1617

[ece38864-bib-0002] Baudry, G. , Hopkins, J. , Watts, P. C. , & Kaitala, A. (2021). Female sexual signaling in a capital breeder, the european glow‐worm *Lampyris noctiluca* . Journal of Insect Behavior, 34(1), 16–25. 10.1007/s10905-020-09763-9

[ece38864-bib-0003] Birkhead, T. , & Hunter, F. (1990). Mechanisms of sperm competition. Trends in Ecology & Evolution, 5(2), 48–52. 10.1016/0169-5347(90)90047-H 21232320

[ece38864-bib-0073] Blanco, C. A. , Perera, O. P. , Groot, A. , Hernández, G. , & Terán‐Vargas, A. P. (2008). Paternity allocation in a mutant heliothis virescens1 colony. Southwestern Entomologist, 33(4), 253–263. 10.3958/0147-1724-33.4.253

[ece38864-bib-0004] Blanco, C. A. , Rojas, M. G. , Groot, A. T. , Morales‐Ramos, J. , & Abel, C. A. (2009). Size and chemical composition of *Heliothis virescens* (Lepidoptera: Noctuidae) spermatophores. Annals of the Entomological Society of America, 102(4), 629–637.

[ece38864-bib-0005] Blankers, T. , Lievers, R. , Plata, C. , van Wijk, M. , van Veldhuizen, D. , & Groot, A. T. (2021). Sex pheromone signal and stability covary with fitness. bioRxiv. 10.1098/rsos.210180 PMC8242834 34234954

[ece38864-bib-0006] Brady, U. E. , & Smithwick, E. B. (1968). Production and release of sex attractant by the female Indian‐meal moth, *Plodia interpu*nctella. Annals of the Entomological Society of America, 61(5), 1260–1265.

[ece38864-bib-0007] Brooks, R. , & Endler, J. A. (2001). Female guppies agree to differ: Phenotypic and genetic variation in mate‐choice behavior and the consequences for sexual selection. Evolution, 55(8), 1644–1655. 10.1111/j.0014-3820.2001.tb00684.x 11580024

[ece38864-bib-0008] Brown, W. D. (1997). Female remating and the intensity of female choice in black‐horned tree crickets, Oecanthus Nigricornis. Behavioral Ecology, 8(1), 66–74.

[ece38864-bib-0009] Burton, R. L. (1970). A low‐cost artificial diet for the corn earworm. Journal of Economic Entomology, 63(6), 1969–1970.

[ece38864-bib-0010] Cotton, S. , Small, J. , & Pomiankowski, A. (2006). Sexual selection and condition‐dependent mate preferences. Current Biology, 16(17), R755–R765. 10.1016/j.cub.2006.08.022 16950102

[ece38864-bib-0011] De Jong, M. C. , & Sabelis, M. W. (1991). Limits to runaway sexual selection: The wallflower paradox. Journal of Evolutionary Biology, 4(4), 637–655. 10.1046/j.1420-9101.1991.4040637.x

[ece38864-bib-0012] Eberhard, W. (1996). Female control: Sexual selection by cryptic female choice (Vol. 69). Princeton University Press.

[ece38864-bib-0013] Edward, D. A. (2015). The description of mate choice. Behavioral Ecology, 26(2), 301–310. 10.1093/beheco/aru142

[ece38864-bib-0014] Elgert, C. , Lehtonen, T. K. , Kaitala, A. , & Candolin, U. (2021). The duration of artificial light defines sexual signalling in the common glow‐worm. Behavioral Ecology and Sociobiology, 75(11), 1–8. 10.1007/s00265-021-03093-2

[ece38864-bib-0015] Fedina, T. Y. , & Lewis, S. M. (2008). An integrative view of sexual selection in *Tribolium* flour beetles. Biological Reviews, 83(2), 151–171. 10.1111/j.1469-185X.2008.00037.x 18429767

[ece38864-bib-0016] Flint, H. M. , & Kressin, E. L. (1968). Gamma irradiation of the tobacco budworm: Sterilization, competitiveness, and observations on reproductive biology. Journal of Economic Entomology, 61(2), 477–483.

[ece38864-bib-0017] Fox, J. , & Weisberg, S. (2019). An R companion to applied regression (3rd ed.). Sage.

[ece38864-bib-0018] Gabor, C. R. , & Halliday, T. R. (1997). Sequential mate choice by multiply mating smooth newts: Females become more choosy. Behavioral Ecology, 8(2), 162–166. 10.1093/beheco/8.2.162

[ece38864-bib-0019] Gao, K. , van Wijk, M. , Clement, Z. , Egas, M. , & Groot, A. T. (2020). A life‐history perspective on sexual selection in a polygamous species. BMC Evolutionary Biology, 20, 1–10. 10.1186/s12862-020-01618-3 32380947 PMC7206733

[ece38864-bib-0020] Groot, A. T. (2014). Circadian rhythms of sexual activities in moths: A review. Frontiers in Ecology and Evolution, 2, 43. 10.3389/fevo.2014.00043

[ece38864-bib-0021] Halliday, T. (1983). The study of mate choice. In: B. Patrick (Ed.), Mate choice. Cambridge University Press.

[ece38864-bib-0022] Heath, R. , McLaughlin, J. , Proshold, F. , & Teal, P. (1991). Periodicity of female sex pheromone titer and release in *Heliothis subflexa* and *H. vires*cens (Lepidoptera: Noctuidae). Annals of the Entomological Society of America, 84(2), 182–189. 10.1093/aesa/84.2.182

[ece38864-bib-0023] Herberstein, M. , Schneider, J. , & Elgar, M. (2002). Costs of courtship and mating in a sexually cannibalistic orb‐web spider: Female mating strategies and their consequences for males. Behavioral Ecology and Sociobiology, 51(5), 440–446. 10.1007/s00265-002-0460-8

[ece38864-bib-0024] Holveck, M.‐J. , & Riebel, K. (2010). Low‐quality females prefer low‐quality males when choosing a mate. Proceedings of the Royal Society B: Biological Sciences, 277(1678), 153–160. 10.1098/rspb.2009.1222 PMC2842619 19812084

[ece38864-bib-0025] Hopkins, J. , Baudry, G. , Candolin, U. , & Kaitala, A. (2015). I’m sexy and I glow it: Female ornamentation in a nocturnal capital breeder. Biology Letters, 11(10), 20150599. 10.1098/rsbl.2015.0599 26490414 PMC4650175

[ece38864-bib-0026] Hosseini, S. A. , van Wijk, M. , Ke, G. , Goldansaz, S. H. , Schal, C. , & Groot, A. T. (2016). Experimental evidence for chemical mate guarding in a moth. Scientific Reports, 6(1), 1–6. 10.1038/srep38567 27934963 PMC5146913

[ece38864-bib-0027] Jennions, M. D. , & Petrie, M. (1997). Variation in mate choice and mating preferences: A review of causes and consequences. Biological Reviews, 72(2), 283–327. 10.1017/S0006323196005014 9155244

[ece38864-bib-0028] Jennions, M. D. , & Petrie, M. (2000). Why do females mate multiply? A review of the genetic benefits. Biological Reviews, 75(1), 21–64. 10.1017/S0006323199005423 10740892

[ece38864-bib-0029] Judge, K. A. , Tran, K.‐C. , & Gwynne, D. T. (2010). The relative effects of mating status and age on the mating behaviour of female field crickets. Canadian Journal of Zoology, 88(2), 219–223. 10.1139/Z09-139

[ece38864-bib-0030] Kassambara, A. , Kosinski, M. , Biecek, P. , & Fabian, S. (2019). survminer: Drawing Survival Curves Using “ggplot2”.

[ece38864-bib-0031] Kelly, C. D. (2018). The causes and evolutionary consequences of variation in female mate choice in insects: The effects of individual state, genotypes and environments. Current Opinion in Insect Science, 27, 1–8. 10.1016/j.cois.2018.01.010 30025624

[ece38864-bib-0032] Kilmer, J. T. , Fowler‐Finn, K. D. , Gray, D. A. , Höbel, G. , Rebar, D. , Reichert, M. S. , & Rodríguez, R. L. (2017). Describing mate preference functions and other function‐valued traits. Journal of Evolutionary Biology, 30(9), 1658–1673. 10.1111/jeb.13122 28556474

[ece38864-bib-0033] Kingan, T. G. , Thomas‐Laemont, P. A. , & Raina, A. K. (1993). Male accessory gland factors elicit change from ‘virgin’ to ‘mated’ behaviour in the female corn earworm moth *Helicoverpa zea* . Journal of Experimental Biology, 183(1), 61–76. 10.1242/jeb.183.1.61

[ece38864-bib-0034] Kokko, H. , Jennions, M. D. , & Brooks, R. (2006). Unifying and testing models of sexual selection. Annual Review of Ecology Evolution and Systematics, 37, 43–66. 10.1146/annurev.ecolsys.37.091305.110259

[ece38864-bib-0035] Kokko, H. , & Mappes, J. (2005). Sexual selection when fertilization is not guaranteed. Evolution, 59(9), 1876–1885. 10.1111/j.0014-3820.2005.tb01058.x 16261726

[ece38864-bib-0036] LaMunyon, C. W. (2000). Sperm storage by females of the polyandrous noctuid moth *Heliothis virescens* . Animal Behaviour, 59(2), 395–402. 10.1006/anbe.1999.1294 10675262

[ece38864-bib-0037] LaMunyon, C. W. (2001). Determinants of sperm precedence in a noctuid moth *Heliothis virescens*: A role for male age. Ecological Entomology, 26(4), 388–394.

[ece38864-bib-0038] LaMunyon, C. W. , & Eisner, T. (1993). Postcopulatory sexual selection in an arctiid moth (*Utetheisa ornatrix*). Proceedings of the National Academy of Sciences, 90(10), 4689–4692. 10.1073/pnas.90.10.4689 PMC46578 8506319

[ece38864-bib-0039] Lindström, K. , & Lehtonen, T. K. (2013). Mate sampling and choosiness in the sand goby. Proceedings of the Royal Society B: Biological Sciences, 280(1765), 20130983. 10.1098/rspb.2013.0983 PMC3712446 23804620

[ece38864-bib-0040] Löfstedt, C. (1993). Moth pheromone genetics and evolution. Philosophical Transactions of the Royal Society of London B, 340(1292), 167–177.

[ece38864-bib-0041] Maxwell, M. R. , Barry, K. L. , & Johns, P. M. (2010). Examinations of female pheromone use in two praying mantids, *Stagmomantis limbata* and *Tenodera aridifolia sinensis* (Mantodea: Mantidae). Annals of the Entomological Society of America, 103(1), 120–127.

[ece38864-bib-0042] Monti, L. , Lalanne‐Cassou, B. , Lucas, P. , Malosse, C. , & Silvain, J.‐F. (1995). Differences in sex pheromone communication systems of closely related species: *Spodoptera latifascia* (Walker) and *S. descoinsi lalannecassou* & *silvain* (Lepidoptera: Noctuidae). Journal of Chemical Ecology, 21(5), 641–660. 24234256 10.1007/BF02033707

[ece38864-bib-0043] Pair, S. , Laster, M. , & Martin, D. (1977). Hybrid sterility of the tobacco budworm: Effects of alternate sterile and normal matings on fecundity and fertility. Annals of the Entomological Society of America, 70(6), 952–954.

[ece38864-bib-0044] Parker, G. A. (1970). Sperm competition and its evolutionary consequences in the insects. Biological Reviews, 45(4), 525–567. 10.1111/j.1469-185X.1970.tb01176.x

[ece38864-bib-0045] Parker, G. A. , & Birkhead, T. R. (2013). Polyandry: The history of a revolution. Philosophical Transactions of the Royal Society B: Biological Sciences, 368(1613), 20120335. 10.1098/rstb.2012.0335 PMC3576588 23339245

[ece38864-bib-0046] Pashley, D. P. , Hammond, A. M. , & Hardy, T. N. (1992). Reproductive isolating mechanisms in fall armyworm host strains (Lepidoptera: Noctuidae). Annals of the Entomological Society of America, 85(4), 400–405. 10.1093/aesa/85.4.400

[ece38864-bib-0047] Phelan, P. L. (1997). Evolution of mate‐signaling in moths: phylogenetic considerations and predictions from the asymmetric tracking hypothesis. In J. C. Choe , & B. J. Crespi (Eds.), The evolution of mating systems in insects and arachnids (pp. 240–256). Cambridge University Press.

[ece38864-bib-0048] Pitcher, T. E. , Neff, B. D. , Rodd, F. H. , & Rowe, L. (2003). Multiple mating and sequential mate choice in guppies: Females trade up. Proceedings of the Royal Society of London Series B: Biological Sciences, 270(1524), 1623–1629. 10.1098/rspb.2002.2280 12908984 PMC1691420

[ece38864-bib-0049] Pope, M. M. , Gaston, L. , & Baker, T. (1982). Composition, quantification, and periodicity of sex pheromone gland volatiles from individual *Heliothis virescens* females. Journal of Chemical Ecology, 8(7), 1043–1055. 10.1007/BF00987885 24415341

[ece38864-bib-0050] Puurtinen, M. , & Fromhage, L. (2017). Evolution of male and female choice in polyandrous systems. Proceedings of the Royal Society B: Biological Sciences, 284, 1851, 20162174. 10.1098/rspb.2016.2174 PMC5378073 28330914

[ece38864-bib-0051] R Core Team . (2021). R: A language and environment for statistical computing [Internet]. R Foundation for Statistical Computing. https://www.R‐project.org/

[ece38864-bib-0052] Ratterman, N. L. , Rosenthal, G. G. , Carney, G. E. , & Jones, A. G. (2014). Genetic variation and covariation in male attractiveness and female mating preferences in *Drosophila melanogaster* . G3: Genes, Genomes, Genetics, 4(1), 79–88. 24212081 10.1534/g3.113.007468 PMC3887542

[ece38864-bib-0053] Raulston, J. , Snow, J. , Graham, H. , & Lingren, P. (1975). Tobacco budworm: Effect of prior mating and sperm content on the mating behavior of females. Annals of the Entomological Society of America, 68(4), 701–704.

[ece38864-bib-0054] Roelofs, W. L. , Hill, A. S. , Linn, C. E. , Meinwald, J. , Jain, S. C. , Herbert, H. J. , & Smith, R. F. (1982). Sex pheromone of the winter moth, a geometrid with unusually low temperature precopulatory responses. Science, 217(4560), 657–659. 10.1126/science.217.4560.657 17817538

[ece38864-bib-0055] Schöfl, G. , Heckel, D. G. , & Groot, A. (2009). Time‐shifted reproductive behaviours among fall armyworm (Noctuidae: *Spodoptera frugiperda*) host strains: Evidence for differing modes of inheritance. Journal of Evolutionary Biology, 22(7), 1447–1459. 19467132 10.1111/j.1420-9101.2009.01759.x

[ece38864-bib-0056] Sheldon, B. C. (1994). Male phenotype, fertility, and the pursuit of extra‐pair copulations by female birds. Proceedings of the Royal Society of London Series B: Biological Sciences, 257(1348), 25–30.

[ece38864-bib-0057] Simmons, L. W. (2001). Sperm competition and its evolutionary consequences in the insects. Princeton University Press.

[ece38864-bib-0058] Slatyer, R. A. , Mautz, B. S. , Backwell, P. R. , & Jennions, M. D. (2012). Estimating genetic benefits of polyandry from experimental studies: A meta‐analysis. Biological Reviews, 87(1), 1–33. 10.1111/j.1469-185X.2011.00182.x 21545390

[ece38864-bib-0059] South, A. , Stanger‐Hall, K. , Jeng, M.‐L. , & Lewis, S. M. (2011). Correlated evolution of female neoteny and flightlessness with male spermatophore production in fireflies (Coleoptera: Lampyridae). Evolution: International Journal of Organic. Evolution, 65(4), 1099–1113. 10.1111/j.1558-5646.2010.01199.x 21108637

[ece38864-bib-0060] Teal, P. , Byers, J. , & Philogene, B. (1978). Differences in female calling behavior of three interfertile sibling species of *Euxoa* (Lepidoptera: Noctuidae). Annals of the Entomological Society of America, 71(4), 630–634.

[ece38864-bib-0061] Therneau, T. M. (2021). A Package for Survival Analysis in R. https://CRAN.R‐project.org/package=survival

[ece38864-bib-0062] Thornhill, R. (1983). Cryptic female choice and its implications in the scorpionfly *Harpobittacus nigriceps* . The American Naturalist, 122(6), 765–788. 10.1086/284170

[ece38864-bib-0063] Thornhill, R. (1984). Alternative female choice tactics in the scorpionfly *Hylobittacus apicalis* (Mecoptera) and their implications. American Zoologist, 24(2), 367–383.

[ece38864-bib-0064] Tumlinson, J. , Hendricks, D. , Mitchell, E. , Doolittle, R. , & Brennan, M. (1975). Isolation, identification, and synthesis of the sex pheromone of the tobacco budworm. Journal of Chemical Ecology, 1(2), 203–214. 10.1007/BF00987869

[ece38864-bib-0065] Vahed, K. (1998). The function of nuptial feeding in insects: A review of empirical studies. Biological Reviews, 73(1), 43–78. 10.1017/S0006323197005112

[ece38864-bib-0066] Wickham, H. (2016). Programming with ggplot2. In ggplot2 (pp. 241–253). Springer.

[ece38864-bib-0067] Wickman, P.‐O. , & Rutowski, R. L. (1999). The evolution of mating dispersion in insects. Oikos, 84(3), 463. 10.2307/3546425

[ece38864-bib-0068] Widemo, F. , & Sæther, S. A. (1999). Beauty is in the eye of the beholder: Causes and consequences of variation in mating preferences. Trends in Ecology & Evolution, 14(1), 26–31. 10.1016/S0169-5347(98)01531-6 10234244

[ece38864-bib-0069] Wong, J. W. , Palaniswamy, P. , Underbill, E. , Steck, W. , & Chisholm, M. (1984). Novel sex pheromone components from the fall cankerworm moth, Alsophila pometaria. Journal of Chemical Ecology, 10(3), 463–473. 10.1007/BF00988092 24318551

[ece38864-bib-0070] Worthington, A. M. , & Kelly, C. D. (2016). Females gain survival benefits from immune‐boosting ejaculates. Evolution, 70(4), 928–933. 10.1111/evo.12890 26920335

[ece38864-bib-0071] Zeh, J. A. , & Zeh, D. W. (2001). Reproductive mode and the genetic benefits of polyandry. Animal Behaviour, 61(6), 1051–1063. 10.1006/anbe.2000.1705

[ece38864-bib-0072] Zweerus, N. L. , van Wijk, M. , Schal, C. , & Groot, A. T. (2021). Experimental evidence for female mate choice in a noctuid moth. Animal Behaviour, 179, 1–13. 10.1016/j.anbehav.2021.06.022

